# Lavender and the Nervous System

**DOI:** 10.1155/2013/681304

**Published:** 2013-03-14

**Authors:** Peir Hossein Koulivand, Maryam Khaleghi Ghadiri, Ali Gorji

**Affiliations:** ^1^Shefa Neuroscience Research Center, Tehran 1996835911, Iran; ^2^Klinik und Poliklinik für Neurochirurgie, Universitätsklinikum Münster, 48149 Münster, Germany; ^3^Razavi Neuroscience Research Center, Mashhad 9198613636, Iran; ^4^Epilepsy Research Center, Westfälische Wilhelms-Universität Münster, 48149 Münster, Germany; ^5^Institut für Physiologie I, Westfälische Wilhelms-Universität Münster, 48149 Münster, Germany; ^6^Department of Neurology, 48149 Münster, Germany

## Abstract

Lavender is traditionally alleged to have a variety of therapeutic and curative properties, ranging from inducing relaxation to treating parasitic infections, burns, insect bites, and spasm. There is growing evidence suggesting that lavender oil may be an effective medicament in treatment of several neurological disorders. Several animal and human investigations suggest anxiolytic, mood stabilizer, sedative, analgesic, and anticonvulsive and neuroprotective properties for lavender. These studies raised the possibility of revival of lavender therapeutic efficacy in neurological disorders. In this paper, a survey on current experimental and clinical state of knowledge about the effect of lavender on the nervous system is given.

## 1. Introduction

The genus *Lavandula* is native to the lands surrounding the Mediterranean Sea and southern Europe through northern and eastern Africa and Middle Eastern countries to southwest Asia and southeast India. It includes more than 30 species, dozens of subspecies, and hundreds of hybrids and selected cultivars. 

The different varieties of this plant range in height from 9 inches to 3 feet, although some may grow taller with age. Lavender are divided into four main categories: *L. angustifolia*, commonly known as English Lavender, is a frost hardy species that has many pretty cultivars, habit, and blossom color (formerly known as *L. vera* or *L. officinalis*); *L. stoechas* is a large plant with greenish-grey foliage and late blooming with a very strong odor (sometimes known as French lavender); *L. latifolia*, a Mediterranean grass-like lavender; and *L. intermedia*, which is a sterile cross between *L. latifolia* and *L. angustifolia*. The various lavenders have similar ethnobotanical properties and major chemical constituents [1].

The main constituents of lavender are linalool, linalyl acetate, 1,8-cineole *B*-ocimene, terpinen-4-ol, and camphor. However, the relative level of each of these constituents varies in different species [1, 2]. Lavender oil, obtained from the flowers of *Lavandula angustifolia* (Family: Lamiaceae) by steam distillation, is chiefly composed of linalyl acetate (3,7-dimethyl-1,6-octadien-3yl acetate), linalool (3,7-dimethylocta-1,6-dien-3-ol), lavandulol, 1,8-cineole, lavandulyl acetate, and camphor. Whole lavender oil and its major components linalool and linalyl acetate are used in aromatherapy. The major components of lavender oil were identified as 51% linalyl acetate and 35% linalool measured by gas chromatography and gas chromatography-linked Fourier Transform Infrared analysis [1–3].

Most commonly lavender is recommended for oral administration. However, it is also being employed in aromatherapy (inhalation of lavender; [4, 5]), aromatherapy massage, dripping oil [6], and bathing [7]. Unlike many other essential oils used in aromatherapy, lavender oil is often applied undiluted to the skin. The study of Jager et al. [8] suggested that essential oils and their components are rapidly absorbed through the skin. Linalool and linalyl acetate were shown to be rapidly detected in plasma after topical application with massage, reaching peak levels after approximately 19 min [8]. At least since medieval periods, lavender has been a source of drugs as well as perfumes, soaps, flavorings, and crafts. Lavender has a long history of medicinal use and is suggested to possess anticonvulsant, antidepressive, anxiolytic, sedative, and calming properties [1, 9–12]. Lavender also prescribed by some medieval physicians such as Ebn-e-sina and Razi for treatment of epilepsy and migraine attacks. Furthermore, lavender is considered beneficial in treatment of pain and tremor [9–12].

In recent years, several animal and human investigations have indeed evaluated traditional medical remedies of lavender using modern scientific methods. These studies raised the possibility of revival of lavender therapeutic efficacy in neurological disorders on the basis of evidence-based medicine [12, 13]. 

## 2. Animal Studies

Several animal experiments suggest anxiolytic, sedative, analgesic, and anticonvulsive and neuroprotective properties for lavender [14]. It was shown that lavender possesses an anticonflict effect in mice [15]. Continuous exposures to lavender essential oils for 7 days significantly inhibited anxiety- and depression-like behaviors tested by elevated plus-maze and forced swimming tests in rats [16]. Lavender oil produced significant antianxiety effects in the Geller conflict and the Vogel conflict tests in mice. Linalool, a major constituent of lavender oil, produced significant anticonflict effects in the Geller and Vogel tests; findings that were similar to those of lavender oil [17]. Effects of lavender oil were compared with chlordiazepoxide, as a reference anxiolytic, on open-field behavior in rats. Lavender oil exhibited antianxiety properties similar to those of chlordiazepoxide [18]. Anxiolytic effect of lavender was also compared with diazepam in elevated plus-maze test in the Mongolian gerbil. Exposure to lavender odor showed an anxiolytic profile similar to diazepam in female gerbils [19]. Investigation of the effects of inhaled linalool on anxiety, aggressiveness, and social interaction in mice showed anxiolytic properties in the light/dark test, increased social interaction, and decreased aggressive behavior [20].

Local anesthetic effect of lavender and its constituents (linalool and linalyl acetate) is reported in both in vivo and in vitro animal experiments [21]. In the rabbit conjunctival reflex test, treatment with a solution of lavender essential oil as well as with linalyl acetate or linalool induced a dose-dependent enhancement in the number of stimuli necessary to provoke the reflex [21]. The methanolic extract of lavender (200–600 mg/kg) dose-dependently produced sedative effects in mice. This was indicated by the relatively longer time for the reestablishment and number of head dips during the traction and hole-board tests [22]. To evaluate the sedative effects of lavender, the immobility of overagitated mice induced by caffeine was ascertained after the inhalation of lavender. Lavender odor significantly increased the immobile state in mice treated with caffeine [23]. Exposure of mice to lavender odor in a dark cage resulted in depression of motor activity, whilst the plasma levels of linalool rose in proportion to the length of exposure [24]. The intraplantar injection of capsaicin produced an intense and short-lived licking/biting response in mice. The capsaicin-induced nociceptive response was reduced significantly by intraplantar injection of lavender and linalool [25]. Either oral administration or inhalation of lavender essential oil significantly reduced the chemical and thermal pain without evidence of central adverse effects in adult mice. Opioidergic neurotransmission seems to be involved in lavender-induced analgesia since only naloxone pretreatment prevents its effect in writhing test. Cholinergic neurotransmitter system also appears to play a role in lavender analgesia. The blockade of muscarinic and nicotinic receptors prevented analgesic effects of lavender [26]. 

Exposure to lavender effectively improved spatial memory deficits induced by dysfunction of the cholinergic system [27]. Administration of lavender in animal model of Alzheimer's disease (rat model established by intracerebroventricular injection of A*β*1) effectively reversed spatial learning deficits [28]. Repeated application of lavender in mice demonstrated a more rapid sleep onset with longer duration of sleep [29]. Anticonvulsant effect for hydroalcoholic extract of lavender was reported against chemoconvulsant-induced seizures in male mice. Lavender inhibited the onset, shortened the duration, and reduced the intensity of seizure attacks [30]. Anticonvulsant effects of lavender together with diminution in spontaneous activity, when combined with other narcotics, have been reported [31, 32]. Inhalation of lavender was also noted to inhibit convulsion induced by pentylenetetrazol, nicotine, or electroshock in mice [33]. Linalool, one of the major components of lavender oil, has been shown to inhibit the convulsion induced by pentylenetetrazol and transcorneal electroshock in different animal models [34, 35], an effect that may induce via a direct interaction with the glutamatergic NMDA subreceptor as well as GABA_A_ receptors [36]. The neuroprotective effect of lavender oil on cerebral ischemia/reperfusion injury was investigated in mice. Focal cerebral ischemia was induced by the intraluminal occlusion. An aqueous extract of lavender has been shown to diminish glutamate-induced neurotoxicity in rat pups cerebellar granular cell culture [37]. Lavender oil significantly decreased neurological deficit scores, infarct size, and the levels of mitochondria-generated reactive oxygen species and attenuated neuronal damage in focal cerebral ischemia induced by the intraluminal occlusion in mice [38].

## 3. Mechanisms of Action of Lavender in the Nervous System

Several investigations were performed to clarify the mechanism of action of lavender in neuronal tissues. Lavender inhibited lipopolysaccharide-induced inflammatory reaction in human monocyte THP-1 cells effect, which might be associated with the expression of HSP70 [39]. Antioxidant and relatively weak cholinergic inhibition was reported for lavender [38, 40] and linalool [41–43]. Linalool inhibited acetylcholine release and alters ion channel function at the neuromuscular junction [44]. These findings indicate that several targets relevant to treatment of Alzheimer's disease; anticholinergic, neuroprotective, and antioxidant activities could be found in lavender. The neuroprotective effect of lavender oil against cerebral ischemia/reperfusion injury is suggested to be attributed to its antioxidant effects [38]. Evaluation of the effects of lavender oil on motor activity and its relationship to dopaminergic neurotransmission revealed that intraperitoneal application of lavender significantly increased rotarod activity and enhanced dopamine receptors subtype D_3_ in the olfactory bulbs of mice [45]. Lavender oil is also suggested to modulate GABAergic neurotransmission, especially on GABA_A_ receptors, and enhance inhibitory tone of the nervous system [29, 36, 46]. Cholinergic system is suggested to play a role in lavender analgesic, antianxiety, antidepression, and anticonvulsant effects of lavender [16, 26, 33].

Fos is a nuclear transcription factor protein encoded by an immediate early gene c-fos, and it is an early marker of neuronal activation. It serves as a transcriptional factor controlling the expression of genes expected to be involved in effective adaptation to certain situations. Lavender oil reduced c-fos expression in paraventricular nucleus of the hypothalamus and dorsomedial hypothalamic nucleus [18]. Lavender oil inhibited dose-dependently the histamine release and anti-DNP IgE-induced tumor necrosis factor-alpha secretion from peritoneal mast cells in mice [47]. It has been shown that lavender oil inhibited the sympathetic nerves innervating the white and brown adipose tissues and adrenal gland and excites the parasympathetic gastric nerve [48, 49]. Odor of lavender oil, and especially its component linalool, affects autonomic nerves probably through a histaminergic response, decreases lipolysis and heat production (energy consumption), and increases appetite and body weight in rats [50]. Lavender may inhibit the sympathetic nerve activity and lipolysis through activation of H_3_-receptors. The hypothalamic suprachiasmatic nucleus and histamine neurons are involved in the lipolytic responses to the lavender oil, and tyrosine phosphorylation of BIT (a brain immunoglobulin-like molecule with tyrosine-based activation motifs, a member of the signal-regulator protein family) is implicated in the relevant signaling pathways [50]. 

## 4. Human Studies

Although there is considerable debate about whether lavender species have a significant clinical potential either alone or as additives to other substances, many human studies support its effectiveness in different neurological and psychological disorders. Lavender was used predominantly in oral administration, aromatherapy, or massage in several clinical studies, and many benefits were claimed for use in such a manner. In addition to psychological effects, aromatherapy is thought to be therapeutically effective due to physiological effects of the inhaled volatile compounds. It is believed that inhaled lavender act via the limbic system, particularly the amygdala and hippocampus [1]. Linalool and linalyl acetate are rapidly absorbed through the skin after topical application with massage and are thought to be able to cause central nervous system depression [8].

### 4.1. Anxiety, Depression, and Lavender

Lavender was used in the treatment of anxiety disorders and related conditions. Three clinical trials were identified which investigated the efficacy of oral lavender oil preparation (silexan; an essential oil produced from lavender flowers by steam distillation), administered once daily at a dose of 80 mg/day, in subsyndromal (mixed) anxiety disorder and generalized anxiety disorder as well as in restlessness and agitation. Anxiolytic effect of lavender was superior to placebo in 221 patients suffering from anxiety disorder. In addition, lavender improved associated symptoms such as restlessness, disturbed sleep, and somatic complaints and had a beneficial influence on general well-being and quality of life [51, 52]. In line with this study, the efficacy of a 6-week-intake of oral lavender oil preparation (Silexan, 80 mg/day), compared to lorazepam, was investigated in adults with generalized anxiety disorder. This study indicates that lavender effectively ameliorates generalized anxiety comparable to 0.5 mg/daily lorazepam [53]. Alleviation of anxiety and mood improvement were reported in thirty-six patients admitted to an intensive care unit, who received lavender oil (diluted to 1% concentration) aromatherapy [54]. The same results were reported for fourteen female patients who were being treated with chronic hemodialysis [55]. A survey in a long-stay neurology in-patient department showed increased mood scores and reduced psychological distress following aromatherapy with lavender accompanied with tea tree and rosemary [56]. An investigation on the effect of lavender aromatherapy (diluted to 2% concentration) on anxiety and depression in the high risk postpartum woman showed a significant improvement of the Edinburgh Postnatal Depression Scale and Generalized Anxiety Disorder Scale after four consecutive weeks of administration of lavender [57]. Lavender odor reduced anxiety in dental patients; however, it has no effect on dental anxiety surrounding thoughts of future dental visits [58, 59]. Testing visual analog scales to assess anxiety, it is suggested that lavender is a simple, low-risk, cost-effective intervention with the potential to improve preoperative anxiety [60]. Orally administered lavender capsules contained 100 or 200 *μ*L of organic *Lavandula angustifolia* oil were tested on responses to anxiety-provoking film clips. In this study, evaluation of State Trait Anxiety Inventory, mood, positive and negative affect scale, heart rate, and galvanic skin response as well as heart rate variation after administration of lavender suggests that lavender has anxiolytic effects in humans suffering from low anxiety, but these effects may not extend to conditions of severe anxiety [61]. A clinical investigation points to antidepressive effect of lavender. Adjuvant therapy of lavender tincture (1 : 5 in 50% alcohol; 60 drops/day) and imipramine (100 mg/daily) in treatment of forty-eight adult outpatients suffering from mild-to-moderate depression led to a better and earlier improvement. Anticholinergic side effects of imipramine, such as dry mouth and urinary retention, were observed less often when lavender administered with impramine. These results suggest that lavender is an effective adjuvant therapy in combination with imipramine, resulting in a superior and quicker improvement in depressive symptoms [62].

### 4.2. Neuroimaging and Lavender

Evaluation of brain regional metabolic activity with positron emission tomography in ten healthy women after the lavender odor stimulus demonstrated neuronal enhancement in the orbitofrontal, posterior cingulate gyrus, brainstem, thalamus, and cerebellum and reduction of activity in the pre/post-central gyrus and frontal eye field. These findings indicate that lavender aromatherapy in addition to relaxation effect may enhance arousal level in some subjects [63]. Using functional magnetic resonance imaging (fMRI), significant activation in major olfactory brain structures, including the primary olfactory cortex, entorhinal cortex, hippocampus and parahippocampal cortex, thalamus, hypothalamus, orbitofrontal cortex, and insular cortex and its extension into the inferior lateral frontal region was reported in nineteen healthy participants after application of 10% lavender diluted in dipropylene glycol [64]. Cortical perfusion increment after sensorial stimulation with lavender was evaluated by single photon emission computed tomography in ten healthy adults. A significant activation was observed in gyrus rectus, orbitofrontal cortex, and superior temporal cortical areas. A slight perfusion increase also existed in middle temporal and parieto-occipital regions [65]. Lavender odor was delivered via the orthonasal (odor perceived through the nose) and retronasal (odor perceived through the mouth) routes and brain response was measured with fMRI in 20 subjects. In addition to the activation at the base of the central sulcus by lavender, retronasal stimulation with odor resulted in a significant peak in the ventral insula compare to orthonasal application. In contrast, orthonasal application yielded a peak in the right caudate nucleus that approached significance in comparison to retronasal way [66].

### 4.3. Electroencephalography (EEG) and Lavender

It has been suggested that some neurological disorders with significant EEG changes, such as epilepsy, may be benefited by aromatherapy [10, 11]. Lavender affects human EEG pattern accompanied with its anxiolytic effect. It is reported that inhalation of lavender (diluted to 10% concentration) for 3 minutes increases alpha power of EEG as decreases anxiety and brings the subject to a better mood in 40 healthy adults [67]. Increases in theta (4–8 Hz) and alpha (8–13 Hz) wave activity may cause a range of general relaxation effects and can be induced by chemical and nonchemical techniques [68]. It has been shown that during inhalation with lavender (diluted to 10% concentration) in 20 participants, the power of theta and alpha wave activities were significantly increased in all brain regions. This study found relaxing effects with increases of alpha wave activities after administering lavender; indicating the EEG evidence of relaxation by lavender aromatherapy [69]. Furthermore, lavender aromatherapy is reported to produces EEG patterns characteristics of subjects' feeling comfortable [70]. Lavender oil administered in an aroma stream shows modest efficacy in the treatment of agitated behavior in patients with severe dementia [71]. 

Resting frontal EEG asymmetry is suggested to be a predictor of symptom change and end-state functioning in patients with social anxiety disorder who undergo efficacious psychological treatment [72]. Evaluation of frontal EEG asymmetry shifting in thirty-nine adult participants and twenty-seven full-term newborns revealed greater relative left frontal EEG activation (associated with greater approach behavior and less depressed affect) after aromatherapy with lavender. Further studies in these volunteers indicate that lavender may induce left frontal EEG shifting in adults and infants, who show greater baselines relative to right frontal EEG activation. It is suggested that both infants and adults with greater relative right frontal EEG activation at baseline may be more affected by lavender application [73]. 

### 4.4. Sleep and Lavender

Lavender has been suggested as an excellent natural remedy to treat insomnia and improve the sleep quality. Single-blind randomized studies investigated the effectiveness of lavender odor on quality of sleep showed that lavender improved the mean scores of sleep quality in fifteen healthy students [74], in sixty-four ischemic heart disease patients [75], and in thirty-four midlife women with insomnia [76]. Ten individuals with insomnia, verified by a score of 5 or more on the Pittsburgh Sleep Quality Index (PSQI), were treated with lavender odor. Six to eight drops of lavender oil added each night to the cartridge improved the PSQI score by −2.5 points. More notable improvements were seen in females and younger participants. Milder insomnia also improved more than severe ones [77]. Oral lavender oil preparation (80 mg/day) showed a significant beneficial influence on quality and duration of sleep and improved general mental and physical health without causing any unwanted sedative or other drug specific effects in 221 patients suffering from subsyndromal (mixed) anxiety disorder [52]. A mixture of essential oils including lavender, basil, juniper, and sweet marjoram is shown to reduce sleep disturbance and improve overall well-being in older patients [78]. In a clinical study on four benzodiazepine dependent geriatric patients, there was a significant decrease in sleep duration by stopping benzodiazepine treatment, which was restored to previous levels by substitution of aromatherapy with lavender oil. This study suggested that ambient lavender oil might be used as a temporary relief from continued medication for insomnia and reduces the side-effects of these drugs [79]. In a study on thirty-one hospitalized patients, administration of lavender odor showed a trend towards an improved quality of daytime wakefulness and more sustained sleep at night [80]. In contrary to these data, it should be noted that the use of aromatherapy massage with lavender oil has no beneficial effect on the sleep patterns of children with autism attending a residential school. It was suggested that this therapy may show greater effects in the home environment or with longer-term interventions [81].

### 4.5. Pain and Lavender

Lavender reported to be useful in the treatment of acute as well as chronic or intractable pain [82]. It has been shown that foot massage using lavender essential oil in 100 ICU patients of whom 50% were receiving artificial ventilation was effective in lowering blood pressure, heart rate, respiratory rate, wakefulness, and pain [83]. Treatment of recurrent aphthous ulceration with lavender oil in 115 patients revealed a significant pain relief mostly from the first dose, ulcer size reduction, increased rate of mucosal repair, and healing within three days of treatment compared to baseline and placebo groups [84]. Stress level, the bispectral index (a promising parameter for monitoring sedation), and pain intensity of needle insertion were significantly reduced after receiving oxygen with a face mask coated with lavender oil for five minutes compared with the control in thirty volunteers [85]. Aromatic oil massage with essential oils blended with lavender, clary sage, and marjoram in a 2 : 1 : 1 ratio in forty-eight outpatients with primary dysmenorrhea alleviated the pain and reduced the duration of dysmenorrhea [86]. Aromatherapy by using lavender essence was also reported as a successful and safe complementary therapy in reduction of pain after the cesarean section in 200 term pregnant women [87] and after episiotomy in 60 primiparous women [88] as well as in perineal discomfort following normal childbirth in 635 women [89, 90]. It has been shown that lavender aromatherapy through an oxygen face mask with two drops of 2% lavender oil can be used to reduce the demand for opioids in twenty-five patients after immediate postoperative period of breast biopsy surgery [91] and for other analgesics in fifty-four patients undergoing laparoscopic adjustable gastric banding [92]. In contrast to these observations, the aroma of essential oil of lavender ease anxiety but not perception of pain during elective cosmetic facial injections of botulinum toxin for the correction of glabellar wrinkle [93]. A course of eight-session manual acupressure with lavender oil (3% lavender oil; used as the massage lubricant) over a three-week period in patients with nonspecific subacute neck pain (32 patients) or low back pain (61 patients) significantly alleviated the neck and back pain and improved movements of the cervical and lumbar spine [94, 95]. Inhalation of lavender essential oil is suggested to be an effective and safe treatment modality in acute management of migraine headaches. Forty-seven patients suffering from migraine attacks reported significant reduction of pain severity and associated symptoms after fifteen minutes inhalation of lavender oil (2-3 drops of the lavender essential oil rubbed onto their upper lip) in the early stages of the attacks [5]. Aromatherapy massage with lavender accompanied with rose geranium, rose, and jasmine in almond and primrose oils once a week for 8 weeks is reported as an effective treatment of menopausal symptoms such as hot flushes, depression, and pain in climacteric women [96].

### 4.6. Cognition and Lavender

The use of aromas to modulate affect and mood has been reported by several ancient and medieval physicians [9–12]. The positive effects of different medicinal plants as cognition enhancers have been reported [97]. To assess the olfactory impact of the essential oils of lavender on cognitive performance and mood in healthy volunteers, the Cognitive Drug Research computerized cognitive assessment battery was performed in 144 participants. Analysis of performance revealed that lavender odor (four drops of oil were applied to a diffuser pad) produced a significant decrement in performance of working memory as well as impaired reaction times for both memory and attention. In addition, a significant effect was found for lavender compared to controls for degree of contentedness, indicating that lavender is capable of elevating mood, or at least maintaining good mood during the completion of a challenging test battery under laboratory conditions [98]. There is an improvement of emotional state in the work environment following the use of the lavender oil burners. Using lavender oil in burners for a 3-month period, nearly 90% of respondents (a total of 66 subjects) believed that there had been an improvement in the work environment following the use of lavender oil [99]. Aromatherapy consisted of the use of rosemary and lemon essential oils in the morning, and lavender and orange in the evening showed significant improvement in personal orientation related to cognitive function in 28 elderly patients suffering from different forms of dementia [100]. It has been shown that unconscious perception of lavender odor can significantly affect the rate of errors made in the mathematical and letter counting tests. In the presence of the odor of lavender, 108 subjects made fewer errors than in the presence of no odor or the odor of jasmine [101]. By comparison, it has been reported lavender to impair arithmetic reasoning, but not memory, when compared to cloves, with no concomitant effect on mood for either odor [102]. Application of oral lavender (80 mg/day) for six weeks in fifty patients suffering from neurasthenia or post-traumatic stress disorder showed significant improvements of their general mental health status and quality of life [103].

## 5. Safety

Although sufficient evidence exists to recommend lavender for short-term treatment of some neurological disorders, long-term trials and observational studies are needed to establish the safety of long-term use as well as overall efficacy in the context of treatment and management of these diseases. The available data suggests that short-term therapy with lavender is relatively safe. However, there are some reports of adverse effects after application of lavender. Gynecomastia coincided with the topical application of products, which contained lavender and tea tree oils was reported in three boys aged between 7 to 10 years. Gynecomastia resolved in all patients shortly after discontinuation of products containing these oils. Furthermore, studies in human cell lines indicated that the lavender oil had estrogenic and antiandrogenic activities [104]. Lavender should be also used cautiously or avoided in patients with known allergy to lavender [105, 106]. In the oral lavender trials, Kasper et al. [52] reported slightly more adverse events in the lavender group than the placebo group; the most frequently reported adverse effects were related to infections and infestations, followed by gastrointestinal disorders and nervous system disorders. Woelk and Schläfke [107] reported slightly more adverse events in the lavender group than the lorazepam group but again none were described as serious. Gastrointestinal adverse events, such as nausea and dyspepsia, after receiving silexan were reported [107]. Ingestion should be avoided during pregnancy (due to emmenagogue effects) [108] and breastfeeding. Lavender oil has no potential for drug abuse [109]. 

## 6. Critical Overview and Conclusion

A recent increase in the popularity of alternative medicine and natural products has renewed interest in lavender and their essential oils as potential natural remedies [2]. This review may be useful to increase our knowledge of lavender pharmacological effects and improve our future experimental and clinical research plans. Although it is shown that lavender may have a significant clinical potential either in their own right or as adjuvant therapy in different disorders, however, due to some issues, such as methodological inadequacies, small sample sizes, short duration of lavender application, lack of information regarding dose rationale, variation between efficacy and effectiveness trials, variability of administration methods, the absence of a placebo comparator, or lack of control groups more standard experiments and researches are needed to confirm the beneficial effect of lavender in the neurological disorders [109]. Methodological and oil identification problems have also hampered the evaluation of the therapeutic significance of some of the research on lavender. The dried lavender flowers used in some trials were sourced from a local herb store (i.e., [62]). Although taxonomic identification was confirmed in these studies, without quantification of key constituents the quality of the herbal product may be questionable [110]. Although some studies defined the contents of lavender, it is essential that all future clinical studies specify the exact derivation of the oils used in the study and, preferably, include a profile of the liquid or the percentage composition of the major constituents. In addition, several factors, such as temperature, skin type and quality, and the size of area being treated, which may affect the level and rate of lavender absorption after massage or aromatherapy, were not considered in several investigations. Many discreet compounds in lavender oil have shown a myriad of potential therapeutic effects, and researchers continue to seek novel treatments to different ailments [2]. 

Only few clinical investigations on lavender are available using diverse administration methods (i.e., oral, aromatherapy, and as a massage oil). The evidence for oral lavender is promising; however, until independent studies emerge with long-term follow-up data, it remains inconclusive [109]. The use of more widely used forms of lavender administrations (aromatherapy, inhalation, massage, etc.) is not currently supported by good evidence of efficacy. Future clinical trials, well-reported and adopting rigorous standard methodology, in combination with experimental pharmacological research, would help to clarify the therapeutic value of lavender for neurological and psychological disorders [109, 110]. 

The apparently low reporting of adverse reactions could imply tolerability and safety [110]. However, most studies failed to provide details which may have masked these and the studies only involved small numbers of participants. It is crucial to get good tolerability and safety data for all modes of lavender application. Thus longer-term follow ups would be required especially for oral lavender before it is recommended for treatment of neurological and/or psychological disorders.
